# Orientin Ameliorates LPS-Induced Inflammatory Responses through the Inhibitory of the NF-*κ*B Pathway and NLRP3 Inflammasome

**DOI:** 10.1155/2017/2495496

**Published:** 2017-01-19

**Authors:** Qingfei Xiao, Zhihui Qu, Ying Zhao, Liming Yang, Pujun Gao

**Affiliations:** ^1^Department of Hepatology, The First Hospital of Jilin University, Changchun 130021, China; ^2^Department of Nephrology, The First Hospital of Jilin University, Changchun 130021, China

## Abstract

Inflammation is a complex response to diverse pathological conditions, resulting in negative rather than protective effects when uncontrolled. Orientin (Ori), a flavonoid component isolated from natural plants, possesses abundant properties. Thus, we aimed to discover the potential therapeutic effects of orientin on lipopolysaccharide- (LPS-) induced inflammation in RAW 264.7 cells and the underlying mechanisms. In our studies, we evaluated the effects of Ori on proinflammatory mediator production stimulated by LPS, including tumor necrosis factor- (TNF-) *α*, interleukin- (IL-) 6, IL-18, and IL-1*β*, along with prostaglandin E_2_ (PGE_2_) and NO. Our data indicated that orientin dramatically inhibited the levels of these mediators. Consistent with these results, the expression levels of cyclooxygenase-2 (COX-2) and inducible nitric oxide synthase (iNOS) were also reduced. Further study demonstrated that such inhibitory effects of Ori were due to suppression of the nuclear factor-kappa B (NF-*κ*B) pathway and nucleotide-binding domain- (NOD-) like receptor protein 3 (NLRP3) inflammasome activation, which may contribute to its anti-inflammatory effects. Together, these findings show that Ori may be an effective candidate for ameliorating LPS-induced inflammatory responses.

## 1. Introduction

Inflammation, which occurs with various pathological conditions, is well recognized as a complicated innate immune response that protects host organisms from external injuries and pathogens [1]. However, excessive inflammation also contributes to the development of metabolic disorders and cancers [2, 3]. Sources of inflammation can be diverse, varying from pathogenic infection to tissue damage [4]. Among these causative factors, lipopolysaccharide (LPS), a major component of the bacterial cell wall and a ligand of Toll-like receptor 4 (TLR4), has the ability to activate various inflammation-related cellular responses [5, 6].

Among downstream events of the TLR4 signaling pathway initiated by LPS, the nuclear factor-kappa B (NF-*κ*B) is one of the most powerful proinflammatory transcription factors, and it drives a classical signal transduction pathway and gene regulation [7]. Heterodimers of NF-*κ*B are physiologically linked to a family of inhibitory proteins (I*κ*Bs) and reside in the cytoplasm in an inactive form. When exposed to stimuli, IKK contributes to the phosphorylation and degradation of I*κ*Bs, and then, the latter detach from NF-*κ*B [8]. Subsequently, NF-*κ*B enters the nucleus and initiates the transcription and expression of proinflammatory genes, such as tumor necrosis factor- (TNF-) *α*, interleukin- (IL-) 1*β*, nitric oxide (NO), and cyclooxygenase-2 (COX-2) [9, 10]. These proinflammatory cytokines, which are crucial for numerous immune processes, are majorly regulated by NF-*κ*B. Among them, IL-1*β*, a key proinflammatory cytokine, plays a pivotal role in LPS-driven inflammation [7]. However, the final mature form of IL-1*β* requires proteolytic cleavage associated with activation of the NLRP3 inflammasome [11]. The nucleotide-binding domain- (NOD-) like receptor protein 3 (NLRP3) is a multiprotein complex composed of NOD-like receptor (NLR), the adaptor protein ASC, and caspase-1 [12]. Once initiated by stimuli, for instance LPS, NLRP3 proteins polymerize and bind to the ASC adaptor, which in turn promotes the recruitment and activation of procaspase-1. Then, the inactive precursors of IL-18 and IL-1*β* are proteolytically cleaved by the promotion of caspase-1 [13, 14]. These secretions of these proinflammatory cytokines further exacerbate the inflammatory process.

Considering the detrimental effects of excessive inflammation, the duration and magnitude of inflammation must be exactly controlled, which may be achieved by regulation of the pathways described above. Therefore, the application of anti-inflammatory drugs may have potential therapeutic effects. Orientin (Ori) is a flavonoid component isolated from natural plants, such as* Ocimum sanctum*,* Phyllostachys* species (bamboo leaves) [15]. Over the past decade, it has been suggested to possess abundant properties, such as antioxidant, antiviral, anti-inflammatory, antiglycation, anticancer, and anti-thrombus activities [16–19]. Despite definite anti-inflammatory effects, the underlying mechanism of anti-inflammatory activity in RAW 264.7 cells has not yet been fully elucidated. Accordingly, in the current study, we sought to evaluate whether the anti-inflammatory activity of Ori is achieved by inhibition of the NF-*κ*B pathway and NLRP3 inflammasome activation.

## 2. Materials and Methods

### 2.1. Reagents

Orientin (Ori), purity > 98%, was purchased from Chengdu Pufei De Biotech Co., Ltd. LPS (*Escherichia coli* 055: B5) and DMSO were provided by Sigma-Aldrich (St. Louis, MO, USA). Antibodies against NLRP3, ASC, caspase-1, Lamin B, *β*-actin, iNOS, COX-2, P-I*κ*B, I*κ*B, and P65 were offered by Cell Signaling (Boston, MA, USA). Enzyme-linked immunosorbent assay (ELISA) kits, such as mouse TNF-*α*, IL-6, IL-18, and IL-1*β*, were provided by Santa Cruz Biotechnology (Santa Cruz, CA). NO kit (catalog no. S0021) and PGE_2_ ELISA kit were obtained from Beyotime Institute of Biotechnology (Nanjing, China) and Biolegend (San Diego, CA, USA), respectively. All other reagents were supplied by Sigma-Aldrich (St. Louis, MO, USA), unless specifically stated elsewhere.

### 2.2. Cell Culture

The RAW 264.7 mouse macrophage cell line was provided by the China Cell Line Bank (Beijing, China). The cells were cultured according to previous reports [1, 7].

### 2.3. Cell Viability Assay

Cells were plated in 96-well plates at a density of 2 × 10^4^ cells per well and incubated for 24 h. The cells were incubated with different concentrations of orientin for 24 h. The methylthiazol tetrazolium (MTT) assay, which detects viable cells based on the generation of formazan, was used to determine cell viability. In detail, 20 *μ*L of MTT solution (5 mg/mL) was added to each well, and the cells were incubated for another 4 h at 37°C. Subsequently, the culture medium was discarded carefully, and 150 *μ*L of dimethyl sulfoxide (DMSO) was added to dissolve the formazan crystals. The optical density (OD) values were detected by a microplate reader.

### 2.4. ELISA

Cells were plated into 24-well plates at a density of 5 × 10^5^ cells per well and incubated for 24 h. The cells were stimulated with or without LPS (1 *μ*g/mL) for another 24 h after pretreatment with various concentrations of orientin for 1 h. The cell culture supernatants were collected to measure TNF-*α*, IL-6, IL-18, and IL-1*β* levels by a commercially available ELISA kit in accordance with the manufacturer's instructions. Then, a microplate reader was employed to detect the absorbance of 450 nm in each well.

### 2.5. Determination of NO and PGE_2_ Production

Cells were plated in 96-well plates at a density of 2 × 10^4^ cells per well and incubated for 24 h. After pretreatment with different dosages of Ori for 1 h, cells were exposed to LPS (1 *μ*g/mL) for another 24 h. Nitric oxide in the culture medium was assessed via a commercially available kit based on the Griess reaction. For the measurement of PGE_2_ levels, RAW 264.7 cells were plated in 24-well plates at a density of 5 × 10^5^ cells per well and incubated for 24 h. After pretreatment with various different dosages of Ori for 1 h, cells were treated with LPS (1 *μ*g/mL) for another 24 h. Then, the supernatants were collected to assess the PGE_2_ levels by ELISA.

### 2.6. Activation of the NF-*κ*B Pathway and NLRP3 Inflammasome

Cells were plated into 6-well plates at a density of 1 × 10^6^ cells per well and incubated for 24 h. For activation of the NF-*κ*B pathway, cells were pretreated with orientin for 6 h, followed by exposure to LPS (1 *μ*g/mL) for 30 min. Cell lysates were resuspended in lysis buffer for RNA extraction and Western blot analysis. For NLRP3 inflammasome activation, cells were pretreated with Ori for 1 h, followed by stimulation with LPS (1 *μ*g/mL) for 6 h and ATP (5 mM) for 40 min. Additionally, cell lysates were resuspended in lysis buffer for Western blot analysis.

### 2.7. Preparation of Nuclear and Cytosolic Fractions

As the manufacturer's instructions from an NE-PER Nuclear and Cytoplasmic Extraction Reagents kit described, cells were subjected to extract their nuclear and the cytoplasmic (Pierce Biotechnology, Rockford, IL, USA). All steps were carried out on 4°C or ice at unless stated otherwise.

### 2.8. Total RNA Extraction and qPCR

Total RNA was isolated from cells in using the Trizol reagent and then reverse transcription into cDNA by employing the Prime-Script RT-PCR kit (Takara). The resulting cDNAs were diluted 10-fold for PCR. The primers used were as follows: iNOS, 5′-AC ATC GAC C CG TCC ACA GTAT-3′ and 5′-CA GAG GGG TAG GCT TGT CTC-3′; COX2, 5′-AC ACA CTC TAT CAC TGG CACC-3′ and 5′-T TAG GGC GAA GCG TTT GC-3′; and *β*-actin, 5′-TC TG TGT GGA TTG TGG CT-3′ and 5′-CT GCT TGC TGA TCC ACA TCTG-3′. The cDNA analysis was carried out by quantitative real-time PCR using the SYBR Green I (Sigma) method. Subsequently, the comparative Ct method take advantage of calculating gene expression changes.

### 2.9. Western Blot Analysis

Cells exposed to LPS, with or without orientin pretreatment, were collected. The cells were lysed in RIPA with protease and phosphatase inhibitors for 30 min. Total protein concentrations in the supernatant were assessed using a BCA protein assay kit (Beyotime, China). Equal amounts of protein were loaded and separated onto 10–12% SDS-PAGE gels and then transferred onto a PVDF membrane. Membranes were blocked with 5% defatted milk for 1 h at room temperature, followed by incubation with primary antibodies at 4°C overnight. After washing, the membranes were incubated with horseradish peroxidase-conjugated secondary antibodies for 1 h at room temperature, and protein signals were visualized using ECL Western blotting reagents.

### 2.10. Statistical Analysis

All results are shown as the mean values ± standard error of the mean (SEM) from at least three independent experiments. Comparisons for multiple groups were performed by one-way analysis of variance (ANOVA) followed by Dunnett's post hoc test. *p* < 0.05 or *p* < 0.01 was considered statistically significant.

## 3. Results

### 3.1. Effects of Ori on Cell Viability

**Figure 1:**
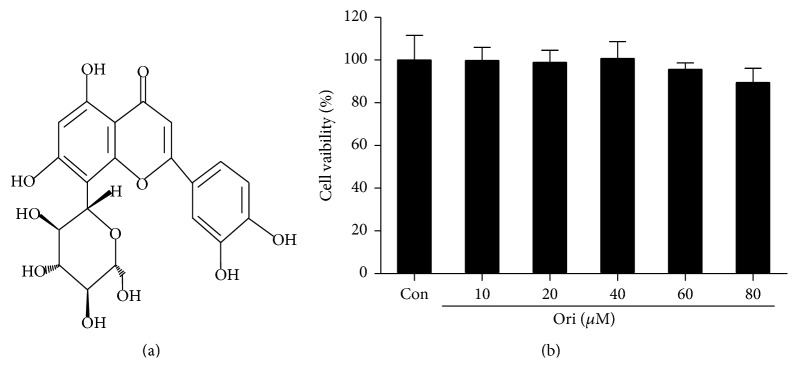
Effects of orientin on the cell viability in LPS-induced in RAW 264.7 cells. (a) The chemical structure of orientin (Ori). (b) Cells were treated with different dosages of Ori (10, 20, 40, 60, or 80 *μ*M) for 24 h and cell viability was evaluated by using an MTT assay.

Whether the cytotoxicity of Ori to RAW 264.7 macrophages was assessed using an MTT assay, the results indicated that Ori did not display any cellular toxicity with concentrations up to 10–40 *μ*M, proving that the inhibitory effects of Ori in LPS-induced inflammation were not due to its cytotoxicity (Figure 1(b)).

### 3.2. Effects of Ori on LPS-Induced Proinflammatory Cytokines

**Figure 2:**
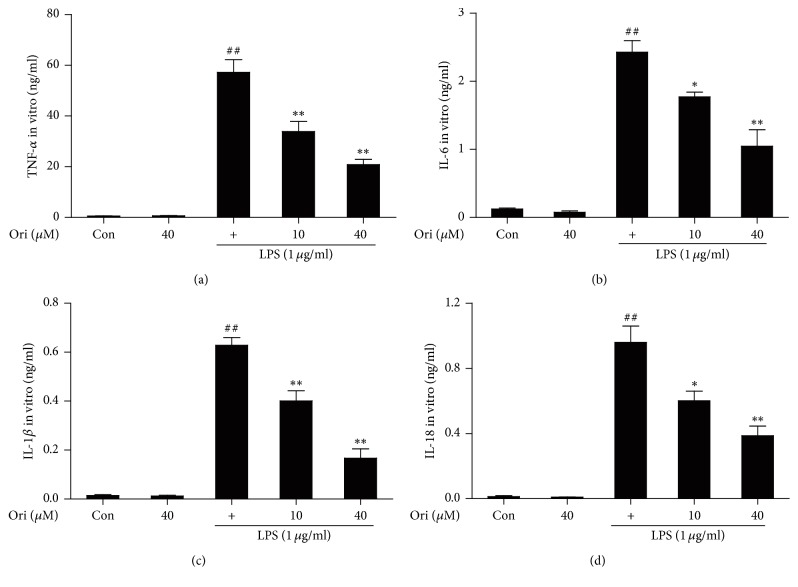
Effects of orientin treatment on the secretion of TNF-*α*, IL-6, IL-1*β*, and IL-18 in LPS-induced RAW 264.7 cells. Cells were pretreated with or without orientin (10 or 40 *μ*M) for 1 h and were then exposed to LPS for another 24 h. (a–d). The effects of orientin on LPS-induced TNF-*α*, IL-6, IL-1*β*, and IL-18 generation were detected by ELISA, respectively. All data of three independent experiments are presented as means ± SEM. ^##^*p* < 0.01 versus the control group; ^*∗*^*p* < 0.05 and ^*∗∗*^*p* < 0.01 versus the LPS group.

To evaluate the anti-inflammatory effects of Ori, proinflammatory cytokines were measured by ELISA. Treatment with LPS alone significantly increased cytokine release in RAW 264.7 cells; however, pretreatment with Ori significantly attenuated release of IL-1*β*, TNF-*α*, IL-6, and IL-18 (Figure 2). These results indicated that Ori inhibited LPS-induced inflammation by reducing the release of proinflammatory cytokines IL-1*β*, TNF-*α*, IL-6, and IL-18 in RAW 264.7 cells.

### 3.3. Effects of Ori on LPS-Induced NO and PGE_2_ Production and iNOS and COX-2 Gene Expression

**Figure 3:**
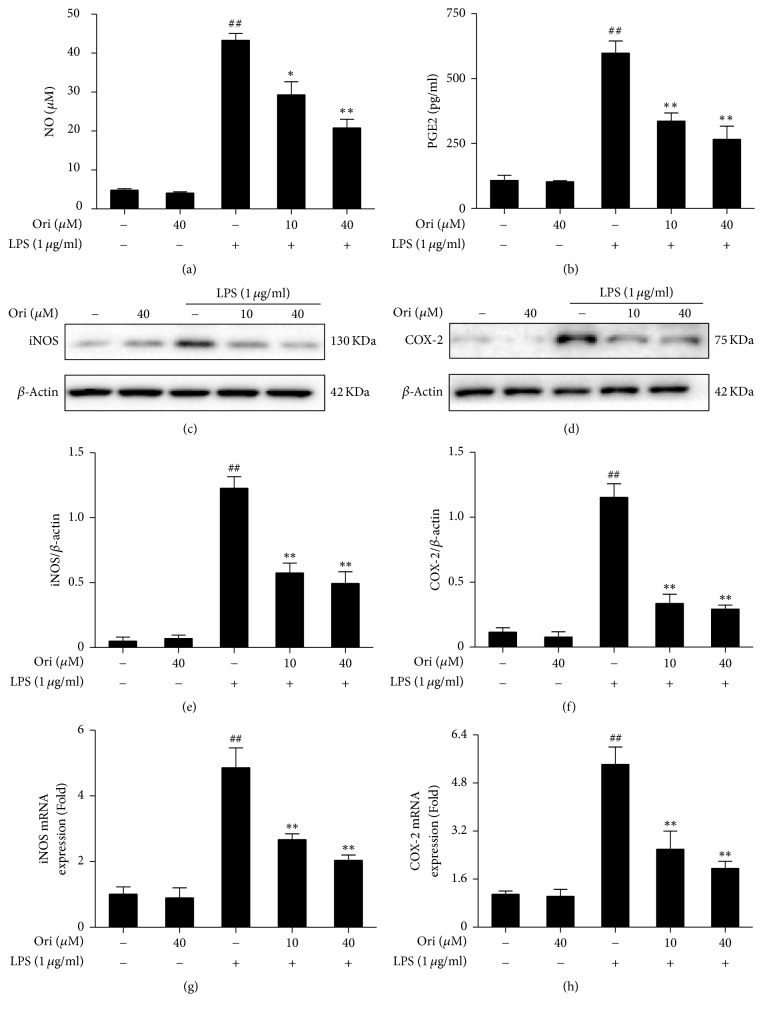
Effects of orientin treatment on LPS-induced NO and PGE_2_ generation, as well as iNOS and COX-2 expression in RAW 264.7 cells. Cells were pretreated with or without Ori (10 or 40 *μ*M) for 1 h and were then exposed to LPS for another 24 h. (a) The effect of Ori on NO production was analyzed by the Griess reaction; (b) PGE_2_ production was measured by ELISA. (c-d) Effects of Ori on LPS-induced iNOS and COX-2 protein expression were analyzed by Western blot. Total RNA was extracted from RAW 264.7 cells and gene expression was quantified using real-time PCR. (g-h) Effects of Ori on LPS-induced iNOS and COX-2 mRNA expression. All data of three independent experiments are presented as means ± SEM. ^##^*p* < 0.01 versus the control group; ^*∗*^*p* < 0.05 and ^*∗∗*^*p* < 0.01 versus the LPS group.

To further clarify the anti-inflammatory effects of Ori, iNOS and COX-2 mRNA and protein expression were also measured. LPS exposure induced markedly increased iNOS and COX-2 mRNA and protein expression, and such changes were blocked by Ori treatment in RAW 264.7 cells (Figures 3(a)–3(d)). Additionally, LPS exposure led to a clear increase in NO and PGE_2_ production, which were suppressed by Ori treatment in RAW 264.7 cells (Figures 3(e)–3(f)). Such results revealed that reduced NO and PGE_2_ production by Ori was due to decreased iNOS and COX-2 expression, which may be important for the inhibition of inflammation.

### 3.4. Effects of Ori on NF-*κ*B (p65) Activation

**Figure 4:**
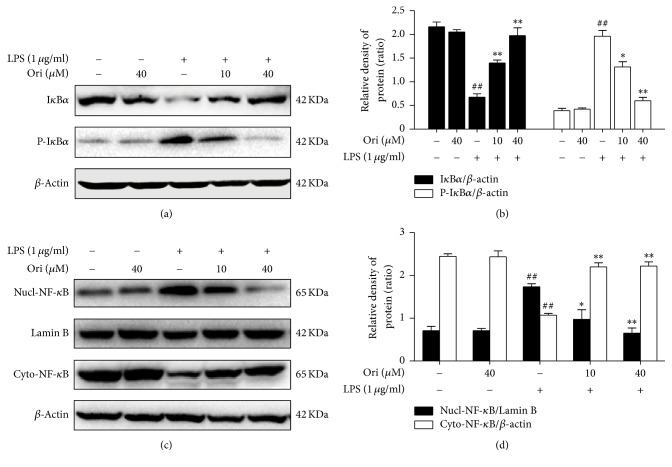
Effects of orientin treatment on LPS-induced the NF-*κ*B activation in RAW 264.7 cells. Cells were pretreated with Ori (10 or 40 *μ*M) for 6 h and then were exposed to LPS for 30 min. The protein levels of I*κ*B*α* and P-I*κ*B*α* as well as the nuclear and cytoplasmic levels of NF-*κ*B (p65) were analyzed by Western blot. The relative densities of protein were performed by densitometric analysis; *β*-actin and Lamin B were used acted as an internal control, respectively. Similar data were repeated in three independent experiments, and one of three representative experiments is shown. All data from three independent experiments are presented as means ± SEM. ^##^*p* < 0.01 versus the control group; ^*∗*^*p* < 0.05 and ^*∗∗*^*p* < 0.01 versus the LPS group.

NF-*κ*B is an essential transcription factor in the expression of proinflammatory cytokines as well as COX-2 and iNOS. To better demonstrate the anti-inflammatory mechanisms of Ori, the effects of Ori on NF-*κ*B activation were examined. The results revealed that Ori pretreatment effectively blocked the phosphorylation and degradation of I*κ*B*α* as well as the nuclear translocation of NF-*κ*B (p65) in LPS-treated RAW 264.7 cells (Figure 4). Thus, the anti-inflammatory effects of Ori might be related to inhibition of the NF-*κ*B pathway.

### 3.5. Effects of Ori on Activation of the NLRP3 Inflammasome

**Figure 5:**
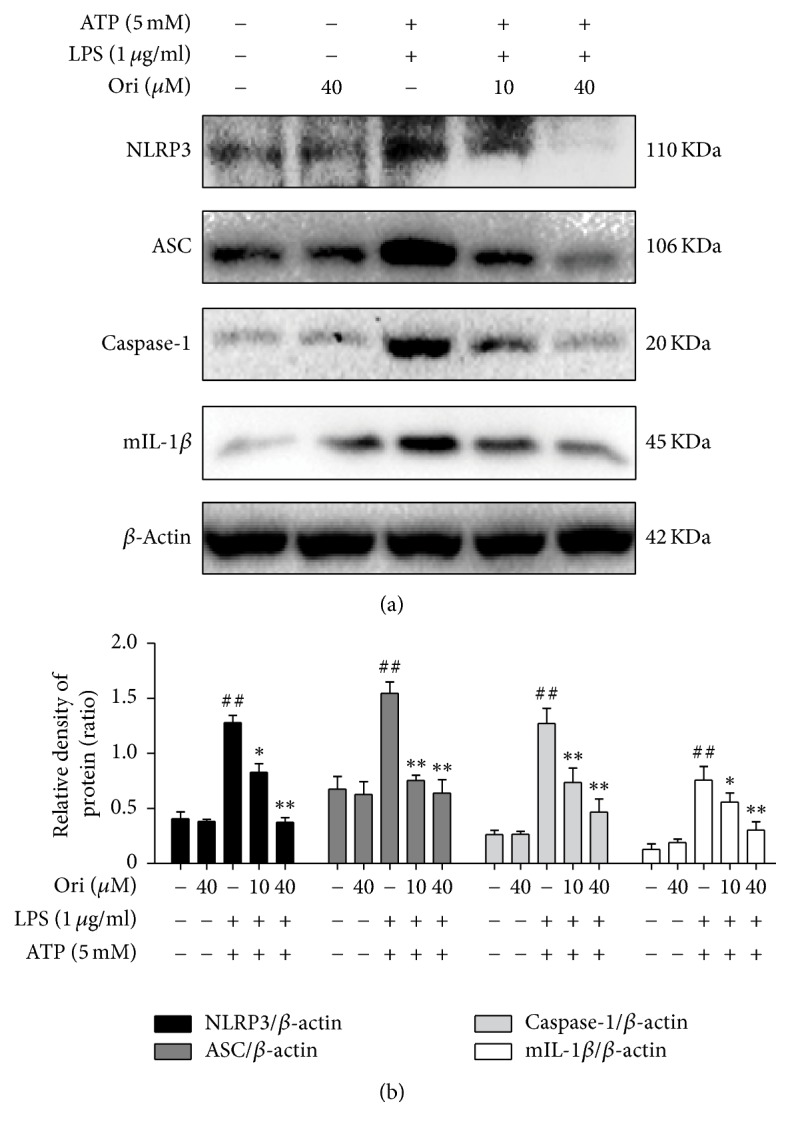
Effects of orientin on the activation of the NLRP3 inflammasome in RAW 264.7 Cells. Cells were pretreated with Ori (10 or 40 *μ*M) for 1 h, followed by stimulation with LPS (1 *μ*g/ml) for 6 h and ATP (5 mM) for 40 min. The protein levels were analyzed by Western blot. The relative densities of protein were performed by densitometric analysis and *β*-actin was used acted as an internal control. Similar data were repeated in three independent experiments, and one of three representative experiments is shown. All data of three independent experiments are presented as means ± SEM. ^##^*p* < 0.01 versus the control group; ^*∗*^*p* < 0.05 and ^*∗∗*^*p* < 0.01 versus the LPS group.

To investigate the effects of Ori on NLRP3 inflammasome activation, Western blot analysis of NLRP3, ASC, and caspase-1 was performed. Treatment of the cells with LPS followed by ATP markedly increased the protein levels of all three components of the inflammasome. However, Ori blocked the increase induced by LPS plus ATP. The result also showed that Ori effectively inhibited IL-1*β* secretion triggered by LPS plus ATP in a dose-dependent manner (Figure 5). Therefore, Ori reduced mature IL-1*β* levels via inhibition of the NLRP3 inflammasome, which is important for inhibiting LPS-induced inflammation.

## 4. Discussion

Inflammation is a complex host response triggered by various pathogens and injury. Despite initial protective effects, it is widely accepted that uncontrolled inflammation promotes further tissue damage and serious disorders [20]. As a major inducer of inflammatory processes, LPS promotes the pathogenesis of inflammation by eliciting vigorous accumulation of neutrophils and the production of inflammatory mediators, including IL-1*β*, TNF-*α*, IL-6, and IL-18 as well as iNOS and COX-2. In previous studies, RAW 264.7 cells treated with lipopolysaccharide were accepted as a canonical model for inflammation research and were applied in our studies [7, 20]. Orientin (Ori), a flavonoid component that has attracted considerable attention because of its anti-inflammatory and antioxidant effects, was explored in the present study to unveil molecular mechanisms of its inhibitory effects on proinflammatory cytokine generation and inflammasome activation in RAW 264.7 cells. Our results revealed that Ori significantly decreased LPS-stimulated production of proinflammatory mediators, such as cytokines IL-1*β*, TNF-*α*, IL-6, and IL-18 as well as iNOS and COX-2. Consistent with this, the levels of iNOS-derived NO and COX-2-derived PGE_2_, which cause inflammation in various diseases, were also remarkably reduced.

In fact, when LPS is recognized by TLR4 as a pathogen-associated molecular pattern (PAMP), it typically activates macrophages via NF-*κ*B. As an essential regulatory transcription factor, NF-*κ*B is implicated in pathways that are involved in the excessive generation of inflammatory mediators [21, 22]. Aberrant NF-*κ*B activity is known to be involved in many inflammatory diseases, including arthritis, sepsis, gastritis, asthma, COPD, and atherosclerosis [23–26]. Therefore, NF-*κ*B has been recognized as a pivotal target for the treatment of inflammation. To elucidate the mechanisms involved in the anti-inflammatory effects of Ori, the impact of orientin on NF-*κ*B activation was examined. As shown in the present study, pretreatment with Ori effectively blocked the phosphorylation and subsequent degradation of I*κ*B and inhibited NF-*κ*B nuclear transcription in LPS-induced RAW 264.7 macrophages. These results indicated that the inhibition of inflammation by Ori might be achieved by suppressing NF-*κ*B pathway activation.

Although proinflammatory cytokines are essential in inflammatory disease and have been proven to be the most important NF-*κ*B targets [23], the TLR4/NF-*κ*B pathway is not sufficient to affect some of them, such as IL-1*β* and IL-18. An increasing number of studies have discovered that NLR is another vital factor during inflammation, except in the TLR-4/NF-*κ*B signaling pathway [27]. Inflammasomes containing different NLRP family proteins have already been identified, such as NLRP1, NLRP3 and NLRC4 [13]. Among them, the NLRP3 inflammasome has been extensively studied in recent years and has been proven to play a pivotal role in the LPS-challenged inflammatory condition. Major components of the NLRP3 inflammasome are composed of three sections: NOD-like receptor family, the pyrin domain containing protein (NLRP), and apoptosis-associated speck-like protein containing a caspase recruitment domain (ASC) and caspase-1. In response to different stimuli, the NLRP3 inflammasome is activated, and ASC then recruits and activates caspase-1. Activated caspase-1 in turn converts pro-IL-1*β* to its mature form [11, 28]. Our results revealed increased expression of NLRP3 inflammasome components in RAW 264.7 cells following treatment with LPS. However, such an increase of the NLRP3 inflammasome and the production of IL-1*β* were abolished by Ori pretreatment.

**Figure 6:**
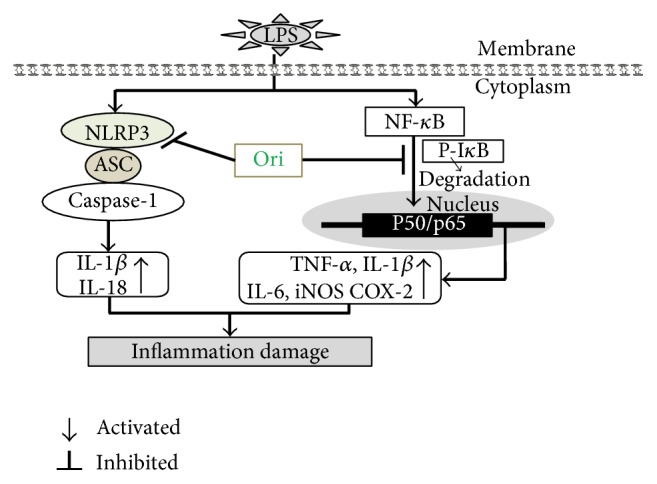
Scheme summarizing the inhibitory of LPS-induced inflammation damage by Ori via suppressing activation of NF-*κ*B pathway and the NLRP3 inflammasome.

In conclusion, as shown in Figure 6, Ori treatment effectively prevented inflammatory damage, which may be closely associated with inhibiting activation of the NF-*κ*B pathway and NLRP3 inflammasome. These results indicated that Ori may be used as a strategy to prevent and treat inflammatory diseases.
